# Design, Synthesis and Multitarget Biological Evaluation of Perfluoroalkylated Benzoylthiourea Compounds: From Biofilm Disruption to DNA Cleavage

**DOI:** 10.1021/acsomega.5c10893

**Published:** 2026-02-09

**Authors:** Mustafa Kemal Yılmaz, Mustafa Kadir Esen, M. Serkan Yalçın, Simay İnce, Sadin Özdemir

**Affiliations:** † Department of Chemistry, Science Faculty, 52983 Mersin University, 33343 Mersin, TÜRKİYE; ‡ Department of Nanotechnology and Advanced Materials, Institute of Science, Mersin University, 33343 Mersin, TÜRKİYE; § Food Processing Program, Technical Science Vocational School, Mersin University, 33343 Mersin, TÜRKİYE; ∥ Department of Chemistry and Chemical Processing Technologies, Technical Science Vocational School, Mersin University, 33343 Mersin, TÜRKİYE

## Abstract

In the present study, a series of benzoylthiourea compounds bearing a perfluorinated group (−C_8_F_17_), namely *N*-((4-(heptadecafluorooctyl)­phenyl)­carbamothioyl)­benzamide (1) and *N*-((3-(heptadecafluorooctyl)­phenyl)­carbamothioyl)­benzamide (2) along with their non-fluorinated analogue, *N*-(phenylcarbamothioyl)­benzamide (3), were synthesized and characterized. Subsequently, various biological properties of the thiourea derivatives 1, 2, and 3 were evaluated, with a particular focus on elucidating the effect of the fluorinated group. The free radical scavenging activities of these compounds were evaluated with ascorbic acid and Trolox standards. Antioxidant activity peaked at 84.56% for 1 and 74.22% for 3. While 1 and 2 showed 97.70 and 96.50% inhibitory effects on α-amylase at 6.25 mg/L, 3 demonstrated 74.90% inhibitory effect at 100 mg/L. All compounds also displayed effective DNA nuclease activity. Additionally, antimicrobial and antibiofilm activities of benzoylthiourea compounds were also investigated. The most resistant microorganisms to the tested compounds were found to be *Escherichia coli* and *Pseudomonas aeruginosa*. In contrast, the most sensitive microorganisms were found to be *Legionella pneumophila* subsp. *pneumophila* and *Enterococcus faecalis*. The biofilm formation inhibition activities of benzoylthiourea compounds against *S. aureus* were 71.79, 69.80, and 63.53%, and against *P*. *aeruginosa* were 53.52, 63.33, and 70.00%, respectively, at the highest concentration. These findings provide a basis for proposing perfluorinated benzoylthiourea derivatives as potential potent, selective, and multitarget medicinal agents.

## Graphical abstract

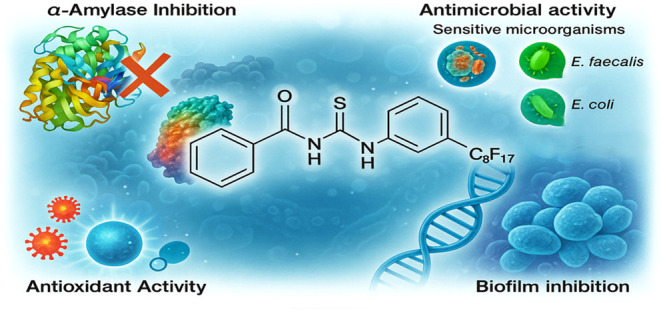

## 1 Introduction

Thioureas or functional thiourea derivatives are known one of the most valuable classes of compounds for various chemical and biological applications, including antiviral, anticancer, antifungal, antidiabetic, antimicrobial, DNA-binding, biofilm, and analgesic properties.^1–7^ Thioureas have emerged as versatile structural motifs in the design of biologically active compounds, primarily due to their NH groups, which act as efficient hydrogen-bond donors. The ability of thioureas to form directional and specific hydrogen bonds with target substrates has also rendered them valuable in a wide range of applications, including biomolecular recognition, enzyme mimetics, and rational drug design. Nonetheless, in functionalized thioureas where a hydrogen atom is replaced by fluorine, it is well-documented that the high bond dissociation energy of the C–F bond (487.5 kJ/mol) and strong electron-withdrawing effect of the fluorine atom confer enhanced molecular stability and interactions.^8^ This substitution significantly modifies the steric and electronic environments of the molecules, which is correlated with enhanced biological activity. For example, fluorine-containing thiourea derivatives were synthesized and evaluated for antidiabetic activity in terms of α-glucosidase inhibition, and it was reported that compounds containing fluorine atoms showed stronger inhibitory activity than non-fluorinated analogues.^9^ In another study, the activity of thiourea structures bearing a fluorine substituent as PPAR-γ agonists was evaluated, and it was demonstrated that compounds containing the fluorine atom may enhance insulin sensitivity and thereby exhibit antidiabetic effects.^10^ Fluorine-containing thiourea derivatives have also been reported to exhibit significant antibacterial and antifungal activities against various pathogenic strains, including *Staphylococcus aureus*, *Escherichia coli*, and *Candida albicans*. The enhanced biological activity of these compounds has been attributed to the strong electron-withdrawing effect of the fluorine atom, which is believed to strengthen the interaction between the molecules and their respective target proteins.^11^

In this context, we report the synthesis of new perfluorooctyl (n-C_8_F_17_) substituted benzoylthiourea compounds (1 and 2) and a non-fluorinated analogue (3) incorporated with known bioactive moieties, as highlighted in Figure 1. To investigate the efficacy, we introduced a perfluorooctyl moiety at the meta and para positions of the phenyl ring while keeping the core structure intact. These compounds were evaluated for DPPH radical scavenging capability, antidiabetic activity, DNA cleavage, antimicrobial activity, and biofilm inhibition studies.

**Figure 1:**
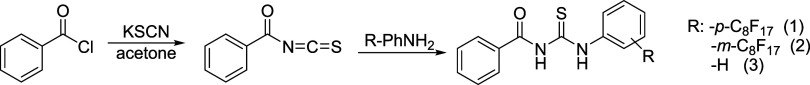
Preparation of benzoylthiourea compounds 1–3.

## 2 Experimental Section

### 2.1 General

Chemicals such as benzoyl chloride, potassium thiocyanate, aniline, 3-heptadecafluorooctyl aniline, and 4-heptadecafluorooctyl aniline were purchased from Sigma-Aldrich and were used without purification. Classical organic solvents (acetone, methanol, ethanol, and dichloromethane, etc.), chemicals, and deuterated NMR solvents (CDCl_3_ and acetone-d6) were also purchased from Sigma-Aldrich. Thin-layer chromatography (TLC) plates (Silica gel 60 coated with fluorescent indicator F254) were used for quick analysis of the reaction’s progress. Melting points (M. p.) of benzoylthioureas were determined by open capillary tubes using a Mettler Toledo MP90 digital melting point apparatus and uncorrected. The characterization of the synthesized benzoylthioureas (1–3) were performed on Bruker Avance 400 Ultrashield Nuclear Magnetic Resonance (^1^H, ^13^C, and ^19^F NMR) and PerkinElmer Spectrum Two/UATR Fourier Transform Infrared (FTIR) spectrometer.

### 2.2 Synthesis of Benzoylthiourea Derivatives

The benzoylthiourea compounds were synthesized according to the previously published method (Figure 1).^12^

#### 2.2.1 *N*-((4-(heptadecafluorooctyl)­phenyl)­carbamothioyl)­benzamide (1)

Solution of benzoyl chloride (0.1 mmol, 11.7 μL) in dry acetone (40 mL) was added dropwise to suspension of potassium thiocyanate (0.1 mmol, 9.52 mg) in acetone (30 mL). The reaction mixture was heated under reflux for 30 min, then cooled to room temperature. Solution of 4-(heptadecafluorooctyl)­aniline (0.1 mmol, 48.6 mg) in acetone (20 mL) was added, and the resulting mixture was stirred for 2 h. Thereafter, the reaction mixture was poured into hydrochloric acid (0.1 N, 300 mL), and the solution was filtered. The solid product was washed with water and purified by recrystallization from ethanol:dichloromethane mixture (1:1, *v*:*v*). Yield: 92% (62.0 mg). White solid. M. p.: 133–134 °C. FT-IR (ATR, ν, cm^–1^): ν­(NH) 3171 (w), ν­(NH) 3047 (w), ν­(C=O) 1667 (s), ν­(C=S) 1518 (s), ν­(C–F, CF_3_) 1193 (s), ν­(C–F, CF_2_) 1142 (s). ^1^H NMR (400.2 MHz, CDCl_3_) δ (ppm) 12.90 (s, 1H, NH), 9.13 (s, 1H, NH), 7.99 (d, *J* = 8.6 Hz, 2H), 7.91 (d, *J* = 7.4 Hz, 2H), 7.61 (dt, *J* = 15.6, 8.4 Hz, 5H). ^13^C NMR (101.6 MHz, CDCl_3_): δ (ppm) 178.3 (s, C=S), 167.1 (s, C=O), 141.0 (s), 134.0 (s), 131.4 (s), 129.3 (s), 127.7 (t, *J*_*FC*_ = 6.6 Hz), 127.5 (s), 123.3 (s). ^19^F NMR (376.5 MHz, CDCl_3_) δ (ppm) −80.72 (t, *J*_*FF*_ = 9.9 Hz, 3F, CF_3_), −110.43 (t, *J*_*FF*_ = 14.5 Hz, 2F, α-CF_2_), −121.15 (bs, 2F, β-CF_2_), −121.69 (bs, 2F, CF_2_), −121.82 (bs, 4F, CF_2_), −122.64 (bs, 2F, CF_2_), −126.04 (bs, 2F, CF_2_).

#### 2.2.2 *N*-((3-(heptadecafluorooctyl)­phenyl)­carbamothioyl)­benzamide (2)

Yield: 94% (63.4 mg). White solid. M. p.: 147–148 °C. FT-IR (ATR, ν, cm^–1^): ν­(NH) 3354 (w), ν­(CH) 2925 (w), ν­(C=O) 1658 (s), ν­(C=S) 1527 (s), ν­(C–F, CF_3_) 1195 (s), ν­(C–F, CF_2_) 1147 (s). ^1^H NMR (400.2 MHz, Acetone-d6) δ (ppm) 9.75 (s, 1H, NH), 8.01 (d, *J* = 8.4 Hz, 2H, ArH), 7.89 (d, *J* = 7.3 Hz, 2H, ArH), 7.56 (d, *J* = 8.5 Hz, 2H, ArH), 7.50–7.46 (m, 1H, ArH), 7.42–7.38 (m, 2H, ArH). ^13^C NMR (101.6 MHz, Acetone-d6): δ (ppm) 166.0 (s, C=O), 143.3 (s), 134.9 (s), 133.5 (s), 131.9 (s), 129.5 (s), 128.8 (s), 128.5 (s), 127.6 (s), 119.9 (s). ^19^F NMR (376.5 MHz, CDCl_3_) δ (ppm) −81.65 (t, *J*_*FF*_ = 9.9 Hz, 3F, CF_3_), −110.27 (t, *J*_*FF*_ = 14.2 Hz, 2F, α-CF_2_), −121.74 (bs, 2F, β-CF_2_), −122.36 (bs, 2F, CF_2_), −123.24 (bs, 6F, CF_2_), −126.72 (bs, 2F, CF_2_).

#### 2.2.3 *N*-(phenylcarbamothioyl)­benzamide (3)

Yield: 94% (24.1 mg). White solid. M. p.: 158–159 °C. FT-IR (ATR, ν, cm^–1^): ν­(NH) 3256 (w), ν­(CH) 2986 (w), ν­(C=O) 1671 (s), ν­(C=S) 1599 (s). ^1^H NMR (400.2 MHz, CDCl_3_) δ (ppm) 12.59 (s, 1H, NH), 9.09 (s, 1H, NH), 7.90 (d, *J* = 7.4 Hz, 2H, ArH), 7.72 (d, *J* = 7.6 Hz, 2H, ArH), 7.69–7.63 (m, 1H, ArH), 7.55 (t, *J* = 7.5 Hz, 2H, ArH), 7.43 (t, *J* = 7.7 Hz, 2H, ArH), 7.32–7.25 (m, 1H, ArH). ^13^C NMR (101.6 MHz, CDCl_3_) δ (ppm) 178.4 (s), 166.9 (s), 137.6 (s), 133.8 (s), 131.7 (s), 129.3 (s), 128.9 (s), 127.5 (s), 127.0 (s), 124.2 (s).

### 2.3 Biological Assays

All biological experiments were conducted in triplicate.

#### 2.3.1 DPPH Radical Scavenging Assay

The method described by Salih Ağırtaş et al. was used with slight changes to assess the antioxidant activity.^13^ The DPPH radical scavenging activity of benzoylthiourea derivatives (1–3) was assessed by mixing 250 μL of each compound at varying concentrations (6.25, 12.5, 25, 50, and 100 mg/L) with 1.0 mL of a 0.002% (w/v) methanolic DPPH solution. Following thorough mixing and a 30 min incubation period at 25 °C in the dark, the resulting color change from deep purple to pale yellow, indicative of radical scavenging, was measured spectrophotometrically at 517 nm. Same procedure used for Trolox and ascorbic acid as standards. A compound-free mixture served as a control. The DPPH radical scavenging activity of the thiourea derivatives was then calculated using the following formula

$$\mathrm{DPP}H\;s\mathrm{cavenging},\;capacity\left(\%\right)=\left(\frac{Abs\left(control\right)-Abs\left(sample\right)}{Abs\left(control\right)}\right)\times 100$$

#### 2.3.2 Antidiabetic Activity

α-Amylase inhibition was performed according to the standard procedure of Oboh et al.^14^ Benzoylthiourea solutions (6.25, 12.5, 25, 50, and 100 mg/L) were mixed with phosphate buffer and α-amylase, then incubated at 37 °C for 15 min. Hydrolysis was initiated by adding 0.2 mL of 1% potato starch solution. After a 20 min incubation at 37 °C, the reaction was terminated with 0.4 mL of 3,5-dinitrosalicylic acid (DNS) and test tubes heated in boiling water for 5 min. A control without benzoylthiourea derivatives was included. Following cooling, the mixtures were diluted with 3 mL of distilled water, and absorbance was measured spectrophotometrically at 540 nm. The antidiabetic activity was then calculated using the formula below.

$$\mathrm{Antidiabetic}\;\mathrm{Activity}\left(\%\right)=\left[(\mathrm{Contro}l_{\mathrm{abs}}-\mathrm{Sampl}e_{\mathrm{abs}}\right)/\mathrm{Contro}l_{\mathrm{abs}}]\times 100$$

#### 2.3.3 DNA Cleavage Activity

DNA agarose gel electrophoresis, a common technique for separating and analyzing DNA, RNA, and proteins by size and charge, was used to assess the nuclease activity of benzoylthiourea derivatives. A 15 μL compound solution and 5 μL pBR322 plasmid DNA were mixed and incubated in the dark at 37 °C for 2 h. Following incubation, the mixture with loading dye was loaded onto a 1% agarose gel (containing 1.0 mg/mL EtBr) in 10× Tris-acetate-EDTA buffer (40 mM Tris-base, 20 mM acetic acid, 1 mM EDTA) and subjected to electrophoresis at 100 V for 1 h. The gel was then visualized using a UV transilluminator.

#### 2.3.4 Antimicrobial Activity

To evaluate the antimicrobial properties of benzoylthiourea compounds, the microdilution technique was utilized. The test microorganisms included *P. aeruginosa* (ATCC 27853), *L. pneumophila* subsp. *pneumophila* (ATCC 33152), *C. glabrata* (ATCC 15126), *S. aureus* (ATCC 29213), *E. coli* (ATCC 35218), *C. krusei* (ATCC 14243), *B. spizizenii* (ATCC 6633) and *E. faecalis* (ATCC 29212). Cultures of these microorganisms were prepared freshly on the day before the assessment. Compounds were diluted in a 1:1 ratio and placed into 96-well microplates for testing. Then microbial inoculations was done and the plates were incubated at 37 °C for 24 h. The minimum inhibitory concentration (MIC) for each compound, which is the lowest concentration required to completely suppress microbial growth, was then determined.

#### 2.3.5 Biofilm Inhibition

The capacity of benzoylthiourea compounds to inhibit biofilm formation was investigated using two bacterial species: *S. aureus* and *P. aeruginosa*. Various concentrations of benzoylthiourea compounds (62.5, 125, and 250 mg/L) were prepared in 24-well plates, and fresh bacterial suspensions were inoculated into wells containing Nutrient Broth (NB) medium. The plates were incubated at 37.5 °C for a period of 72 h to enable the bacteria to adhere to surfaces. Following incubation, the wells containing biofilms were gently rinsed twice with 200 μL of phosphate-buffered saline (PBS) and left to air-dry for half an hour. Subsequently, 200 μL of a 1% aqueous crystal violet solution was introduced to each well to stain the biofilm, allowing the staining process to occur over 60 min. The wells were then rinsed with PBS to remove excess crystal violet. Ethanol was added to the wells and left at ambient temperature for 15 min to extract the absorbed dye. Biofilm inhibition percentages were measured using a spectrophotometer set at 595 nm and calculated using a defined formula.

$$\mathrm{Biofilm}\;\mathrm{inhibition}\left(\%\right)=\left(\frac{\mathrm{Abs}\left(\mathrm{control}\right)-\mathrm{Abs}\left(\mathrm{sample}\right)}{\mathrm{Abs}\left(\mathrm{control}\right)}\right)\times 100$$

All biological assays were performed in triplicate, and the average values were reported.

## 3 Results and Discussion

### 3.1 DPPH Radical Scavenging Activity

Free radicals, reactive molecules with unpaired electrons, are constantly produced in the body and normally scavenged by endogenous antioxidants. However, when the body’s antioxidant defenses are insufficient, external supplementation is needed to prevent damage to vital biomolecules like proteins and DNA.^15^ Thiourea derivatives are known scavengers of O_2_^•–^ and OH^•–^.^16^ The DPPH radical scavenging assay utilizes the 2,2-diphenyl-1-picrylhydrazyl (DPPH.) radical, which forms a purple solution that is reduced by antioxidants via hydrogen atom donation, resulting in yellow-colored diphenylpicrylhydrazine. The absorbance of the DPPH^•^ solution is measured at 517 nm. The results are presented in Figure 2. Increasing the concentration from 50 to 100 mg/L enhanced the DPPH radical scavenging activity of compounds 1, 2, and 3 from 73.22 to 84.56%, 12.20 to 17.80%, and 22.78 to 74.22%, respectively. Ascorbic acid and Trolox used as standards and they exhibited 100% scavenging at 100 mg/L. It was found that compounds 1 and 3 exhibited better antioxidant properties than compound 2, but less than those of Ascorbic acid and Trolox. Compound 1 with para-C_8_F_17_ substituent on the aryl ring was more active than its structurally similar compound 2 that bearing meta-C_8_F_17_ substituent on the aryl ring indicating that para-C_8_F_17_ substituent was more effective for antioxidant activitiy. These results can be generally attributed to the fact that the radical scavenging activity can be significantly changed by introducing different substituents around the aryl ring of thiourea. The number and position of substituents also influence radical scavenging activity.

**Figure 2:**
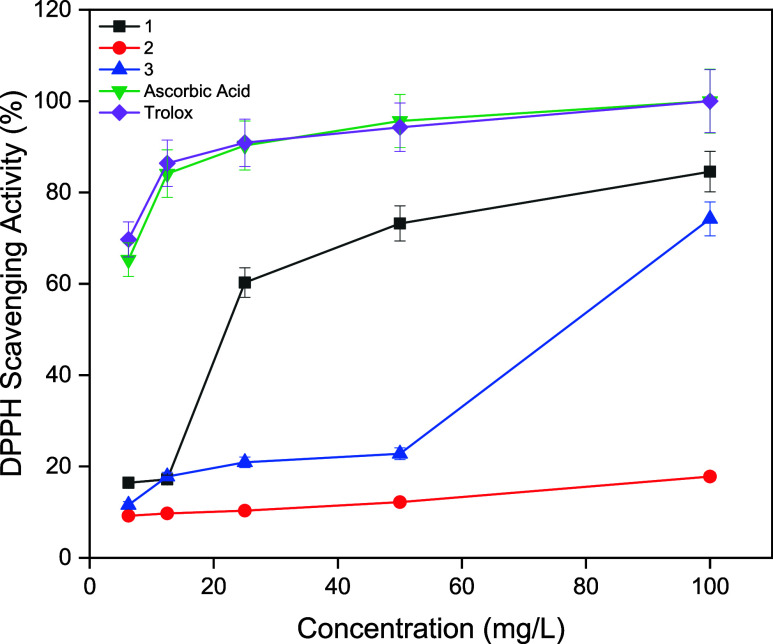
Antioxidant activities of compounds 1–3.

Hussain et al. synthesized oxadiazole-based thiourea derivatives containing the imidazopyridine moiety. They reported that the synthesized analogs showed significant DPPH scavenging activities due to the change in the position of the substituents around the aryl moiety.^17^ Huong et al. reported that 1,3-diphenyl-2-thiourea derivatives demonstrated stronger antioxidant activity than 1-benzyl-3-phenyl-2-thiourea derivatives. Kinetic calculations suggested hydrogen atom transfer as the dominant mechanism over single electron transfer in the reaction of these thiourea derivatives with free radicals.^18^ Nadeem et al. synthesized palladium­(II)-containing thiourea derivatives and tested their antioxidant activity. Among the heterobinuclear complexes formed by treatment with transition metals Zn­(II), Cd­(II), and Co­(II), the complexes containing Co­(II) showed moderate antioxidant activity.^19^ Our results indicate that compounds 1 and 3 are more effective radical scavengers than compound 2, and also, compounds 1 and 3 possess antioxidant potential, warranting further investigation.

### 3.2 Antidiabetic Activity

Diabetes mellitus, a metabolic disorder characterized by impaired glucose regulation, leads to complications like cardiovascular disease, kidney disease, retinopathy, and neuropathy.^20,21^ Inhibiting α-glucosidase and α-amylase to reduce carbohydrate absorption is a therapeutic strategy to manage hyperglycemia. Thioureas, versatile scaffolds with diverse biological activities, are employed in drug design due to their substitutable structures.^22^ Therefore, compounds 1, 2, and 3 were investigated for their inhibitory effects on α-amylase enzyme. The inhibition findings showed that the compounds effectively inhibited α-amylase, depending on the concentration (Figure 3). As the concentration increased from 6.25 to 100 mg/L, the percentage inhibition of compounds 1 and 2 decreased from 97.70 to 50.00%, from 96.50 to 69.90%, respectively, while that of compound 3 increased its antidiabetic ability from 13.70 to 74.90%. These findings indicated that compounds 1 and 2, which exhibited more effective inhibition at low concentrations, are therefore more favorable. On the other hand, 3 also displayed a powerful antidiabetic ability at 100 mg/L. Compound 1 bearing a para-C_8_F_17_ substituent on the aryl ring, and compound 2, bearing a meta-C_8_F_17_ substituent on the aryl ring were found to have a greater inhibitory effect against α-amylase. Although all our compounds have the same basic core, they differ from each other by substitution in their aryl rings, which explains the variation in their inhibition effects. The steric and electronic environments of the molecules are significantly altered by this change, and this is associated with increased biological activity. The strong electron-withdrawing effect of the fluorine atom is believed to be the reason for the increased biological activity by improving the interaction between the molecules and the target protein.

**Figure 3:**
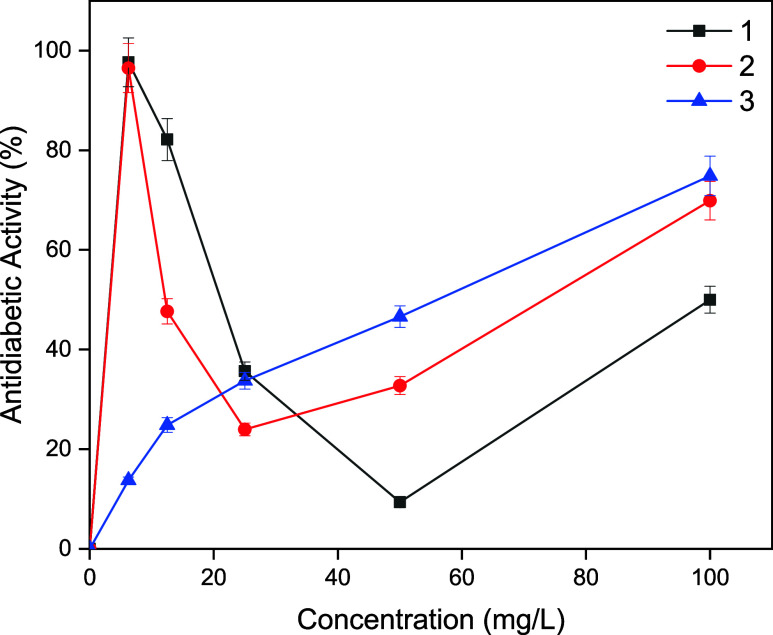
Antidiabetic activities of compounds 1–3.

Nadeem et al. investigated the antidiabetic properties of isatin-based bis-thiourea analogues. They reported that all the analogues showed good α-amylase inhibitory potential.^19^ In another study by Hussain et al., oxadiazole-based thiourea derivatives with an imidazopyridine moiety were synthesized, and their antidiabetic effects were also investigated. They observed that the test compounds exhibited significant α-amylase inhibition.^16^ A study reported by Khan et al. revealed that in terms of antidiabetic properties, the compound 3-(3-(dimethylcarbamoyl)­thioureido) benzoic acid, followed by 3-(3-(diethylcarbamoyl)­thioureido)­benzoic acid was more effective against α-amylase.^23^ After supporting our results with the literature, we can state that all three compounds, especially compounds 1 and 2, have potential as antidiabetic agents.

### 3.3 DNA Cleavage Activity

Gel electrophoresis is a technique used to identify DNA and RNA sequences in organisms, aiding in developing drugs to combat disease. Small organic compounds that bind to DNA can alter its structure and function. Chemical derivatives, among many such binders, demonstrate DNA affinity through various binding modes or cleavage mechanisms. This suggests their potential as antineoplastic agents and their ability to stabilize the topoisomerase II-DNA complex, inducing apoptosis. It functions based on DNA migration in the presence of an electric field.^24^ Gel electrophoresis is used to track the DNA’s transformation from its supercoiled form I (SC) to its nicked circular form II (NC) and linear form III (LC). We investigated the cleavage of DNA strands by compounds 1, 2, and 3 for comparative purposes. The gel electrophoresis image of this study is presented in Figure 4. The absence of any bands in the electrophoresis image at 50, 100, and 200 mg/L concentrations suggests that instead of the DNA transforming from its supercoiled form I (SC) to its nicked circular form II (NC) and linear form III (LC), the DNA was completely fragmented into small oligonucleotides that were not visible in the electrophoresis. Reactive oxygen species (ROS) are short-lived, highly reactive oxygen-containing molecules that can damage DNA and affect the DNA damage response. ROS are known to induce DNA damage, particularly in the context of double-strand breaks. In the literature, DNA denaturation is considered the most effective way to prevent uncontrolled cancer cell proliferation. Anticancer agents cause permanent damage to DNA and promote apoptosis.^25^

**Figure 4:**
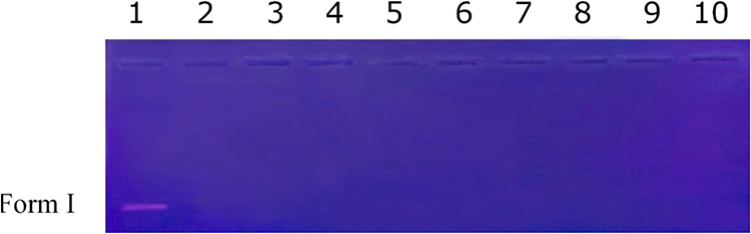
DNA cleavage activities of compounds 1–3. Lane 1, pBR322 DNA; Lane 2, pBR322 DNA + 50 mg/L of 1; Lane 3, pBR322 DNA + 100 mg/L of 1; Lane 4, pBR322 DNA + 200 mg/L of 1; Lane 5, pBR322 DNA + 50 mg/L of 2; Lane 6, pBR322 DNA + 100 mg/L of 2; Lane 7, pBR322 DNA + 200 mg/L of 2; Lane 8, pBR322 DNA + 50 mg/L of 3; Lane 9, pBR322 DNA + 100 mg/L of 3; Lane 10, pBR322 DNA + 200 mg/L of 3.

Yallur et al. synthesized bivalent Cu­(II), Co­(II), and Ni­(II) complexes of [(E)-[(2-methyl-1,3-thiazol-5-yl)­methylidene]­amino]­thiourea, reporting that only the Cu­(II) complex, likely due to deprotonation, exhibited significant DNA cleavage activity in the presence of hydrogen peroxide (cleave SC DNA to the nicked circular (NC) form). The Ni­(II) and Co­(II) complexes showed negligible activity.^24^ Chetana et al. studied the DNA cleavage activity of Cu­(I) complexes containing N,N′-disubstituted thiourea in the presence of hydrogen peroxide. They reported that complexes containing 1-benzyl-3-(4-methyl-pyridin-2-yl)-thiourea and 1-benzyl-3-(6-methyl-pyridin-2-yl)-thiourea cleaved SC DNA to the nicked circular (NC) form.^26^ In conclusion, our DNA cleavage findings suggest that compounds 1, 2, and 3 may serve as potential anticancer agents, warranting further investigation.

### 3.4 Antimicrobial Activity

The increasing resistance to antibacterial drugs represents a significant threat to public heal. The misuse and overuse of traditional antimicrobial medicines have contributed to the emergence of multidrug-resistant bacteria and fungi. This situation highlights the urgent need to develop new antimicrobial agents for treating these resistant microorganisms.^27^ Ureas and thioureas are key scaffolds in medicinal chemistry and constitute fundamental building blocks of a wide range of drugs and bioactive compounds.^28^ Thioureas and their derivatives have long been utilized as therapeutic agents due to their biological activities, including antibacterial and anti-inflammatory effects.^29^ Thiourea compounds are effective against Gram-positive and Gram-negative pathogens through their potent antibacterial effects, which occur through inhibition of cell wall or protein synthesis and possibly disruption of membrane integrity.^28^ Thiazole derivatives have been designed to target many strains that exhibit high resistance. These strains include *E*. species, *A*. *baumannii*, *P*. *aeruginosa*, *E*. *cloacae*, *S*. *aureus*, and *Candida* species. These microorganisms are notable for their resistance to fluconazole and play an important role in the development of various human diseases, such as lung and urinary tract infections.^30^

Table 1 shows the antimicrobial activities of benzoylthiourea compounds obtained by the microdilution method using *P. aeruginosa*, *L. pneumophila* subsp. *pneumophila*, *C. glabrata*, *S. aureus*, *E. coli*, *C. krusei*, *B. spizizenii*, and *E. faecalis* microorganisms. While the most resistant microorganism to compounds 1 and 3 was determined to be *E. coli* (with MIC values of 256 mg/L for 1 and 512 mg/L for 3), the most resistant microorganism to compound 2 was determined to be *P. aeruginosa* (with MIC values of 128 mg/L). The most sensitive microorganisms to compounds 1, 2, and 3 were *L. pneumophila* subsp. *pneumophila* (with MIC values of 16 mg/L for 1 and 2 and 32 mg/L for 3) and *E. faecalis* (with MIC values of 16 mg/L for 1, 8 mg/L for 2, and 32 mg/L for 3). When these values of the compounds were compared, it was seen that the compounds containing perfluorinated group (compounds 1 and 2) had better antimicrobial activity than the nonfluorinated analogue (compound 3).

**Table 1:**
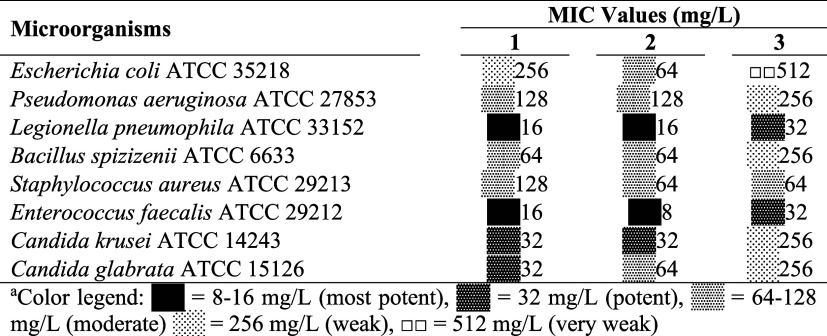
MIC Values of Test Compounds 1–3

In a study evaluating the antibacterial potential of unsymmetrical thiourea derivatives was tested against selected bacterial strains, including *E. coli*, *S. flexneri*, *P. aeruginosa*, and *S. typhi*, none of the compounds showed a strong antimicrobial effect. However, they showed moderate antibacterial activity compared to the standard antibacterial drug Cephradine.^31^ Tagiling et al. synthesized and characterized fluorinated thioureas with various substituents and investigated their antibacterial properties. They found that the compounds inhibited at least two bacterial strains (*B. cereus* and *S. aureus*) in their antibacterial activity, but none of them were effective against *E. coli*.^32^ In a study, the biological activities of newly synthesized tris-thioureas were investigated against Gram-positive bacteria (*S. aureus* and *B. cereus*) and Gram-negative bacteria (*E. coli*). These compounds, which did not show any activity against *P. aeruginosa*, were reported to have strong antibacterial properties against the other mentioned bacteria.^33^ Limban et al. reported the synthesis of benzoylthiourea derivatives and evaluated their antimicrobial potential. It was determined that benzoylthiourea derivatives exhibited good antimicrobial ability especially against *C. albicans* with 2-((4-ethylphenoxy)­methyl)-N-(2,4,6-tri­fluoro­phenyl­carba­mothioyl)­benzamide (5d).^34^

Our study reveals that benzoylthiourea derivatives can be powerful antimicrobial agents against pathogenic microorganisms. The superior antimicrobial effects of these compounds, especially those containing the perfluorinated group, are a valuable guide for future research in this field.

### 3.5 Biofilm Inhibition

Biofilm is a microbial community in which microorganisms are surrounded by an extracellular matrix (EPS) composed of eDNA, proteins, and carbohydrates. Microbes in this structure exhibit higher resistance to antimicrobial agents and host defense mechanisms and are responsible for 60–80% of bacterial infections in humans.^35^ The National Institutes of Health reported that biofilm-related infections are expected to be three times more common than microbial infections in the United States in 2022–2023, causing approximately 36.000 deaths per year. The proliferation of biofilms on both living and nonliving surfaces is a serious health threat. Resistance to these infections is due to the biofilm’s protective structure, which consists of organic matter.^36^ *S. aureus* and *P. aeruginosa* are among the microorganisms that frequently cause hospital infections and have a high biofilm production capacity. These species are included in the ESKAPE (*E*. *faecium*, *S*. *aureus*, *Klebsiella pneumoniae*, *P*. *aeruginosa*, and *Enterobacter* spp.) pathogen group and require urgent and effective treatments because they are multidrug-resistant.^35^ *Pseudomonas* spp. can form biofilms on different surfaces by producing extracellular polymeric substances, and can live together with other pathogens to gain resistance to harsh conditions. *P. aeruginosa* is defined as one of the worst human pathogens based on various virulence factors and mechanisms in infections. It also shows resistance to antibiotics and the immune system by forming biofilms on catheters and prostheses.^37^ *S. aureus* has high pathogenicity and adaptability that can cause serious infections that threaten the immune system. This bacterium, which has developed resistance to various antibiotics, is resistant to treatments with multiple resistance mechanisms, including biofilm production, cell wall changes, DNA changes, efflux pumps, and the presence of enzyme secretions.^36^ Effective methods against bacterial biofilms are limited; therefore, it is of great importance to discover new compounds with antibacterial properties.^38^ Thiourea derivatives attract attention with their antiviral and antibacterial properties, and structural modifications increase their biological activities. These compounds have an important place in medicinal chemistry and drug development.^27^ Thioureas exhibited significant antibacterial effects, particularly targeting Gram-positive strains.^7^ The biofilm inhibition abilities of benzoylthiourea compounds against *S. aureus* and *P. aeruginosa*, two important biofilm-forming bacteria, were investigated. Biofilm inhibition percentage for both microorganisms were calculated and shown in Figures 5 and 6.

**Figure 5:**
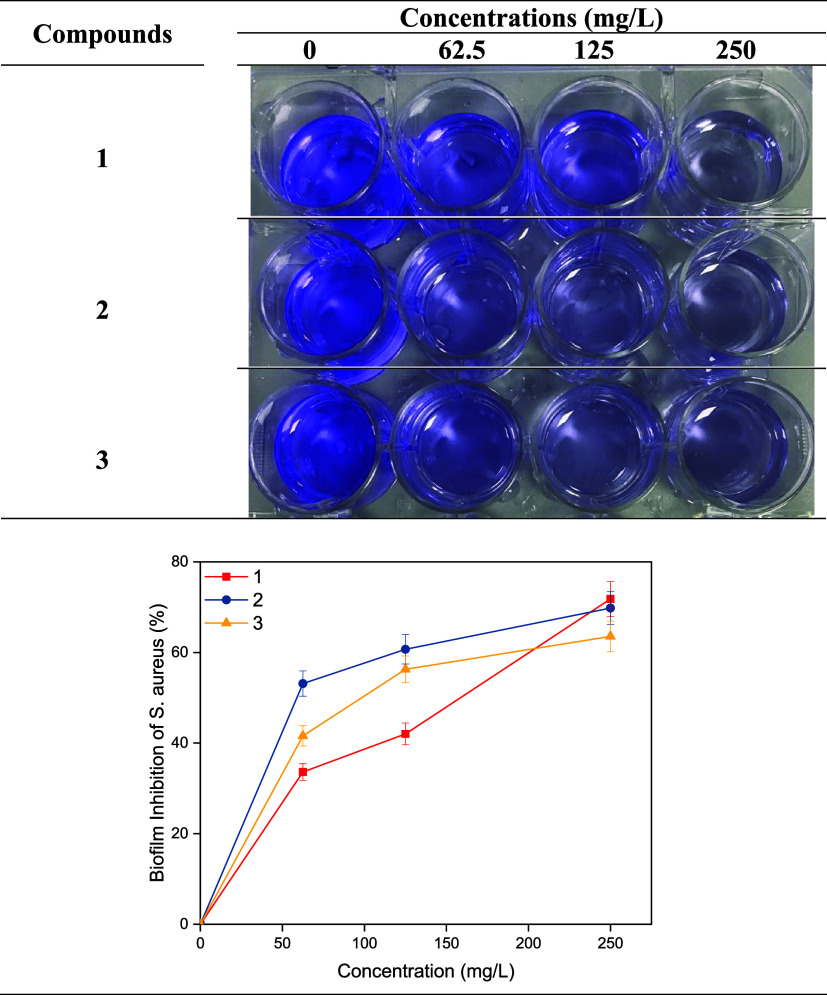
Biofilm inhibition of *S*. *aureus*.

**Figure 6:**
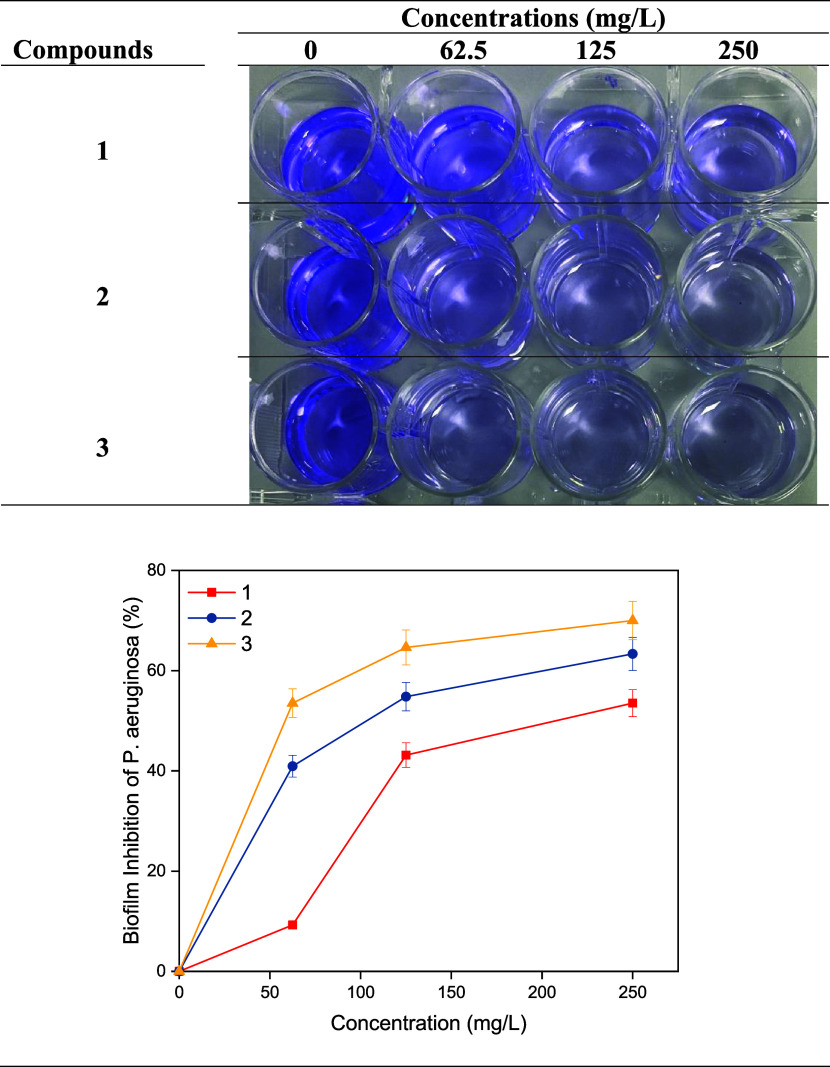
Biofilm inhibition of *P*. *aeruginosa*.

Inhibition percentages of compounds 1, 2, and 3 against *S. aureus* at 62.5 mg/L concentration were found to be 33.62, 53.13, and 41.60%, respectively, while at 250 mg/L concentration, they were found to be 71.79, 69.80, and 63.53%, respectively. The inhibition percentages of compounds 1, 2, and 3 against *P. aeruginosa* at 62.5 mg/L concentration were found to be 9.25, 40.93, and 53.52%, respectively, while they were found to be 53.52, 63.33, and 70.00% at 250 mg/L concentration, respectively. As the concentrations of all compounds increased, their antibiofilm activities against bacteria also increased. While the antibiofilm activity of compounds 1 and 2 containing perfluorinated groups at different positions against *S. aureus* was higher than the non-fluorinated analogue 3, the antibiofilm activity of compound 3 against *P. aeruginosa* was higher. The antibacterial and antibiofilm activities may be attributed to the presence of electron-rich atoms, such as oxygen and sulfur, which allow synthetic thiourea derivatives to interfere with bacterial cell wall structures. In a releated study, in which a series of new thiourea derivatives of 1,3-thiazole were synthesized, it was reported that 3,4-dichlorophenyl and 3-chloro-4-fluorophenyl substituted derivatives effectively inhibited biofilm formation of both methicillin-resistant and standard *S. epidermidis* strains.^39^ Stefaska et al. prepared five thiourea derivatives and reported that they effectively inhibited the biofilm formation of methicillin-resistant and standard *S. epidermidis* strains.^38^ In a study on the synthesis and characterization of new fluoro/trifluoromethyl-substituted acylthiourea derivatives, *in vitro* antimicrobial activities of these compounds against planktonic and biofilm-embedded microbial cells (*P. aeruginosa*, *E. faecalis*, *S. aureus*, *C. albicans*, and *E. coli*) were investigated. The study stated that some compounds exhibited promising antibacterial and antifungal activities with low minimum inhibitory concentrations ranging from 0.15–2.5 mg/mL and minimum biofilm eradication concentrations at 0.019–2.5 mg/mL.^40^ Limban et al. highlighted that certain recently synthesized thiourea derivatives carrying aryl groups substituted with one iodine, bromide, fluorine, or two or three chloride atoms could be used to develop new antimicrobial agents with antibiofilm effects.^41^ In a study in which 31 thiourea derivatives were prepared by reacting 3-(trifluoromethyl)­aniline and commercial aliphatic and aromatic isothiocyanates, it was reported that 1-(3-chloro-4-fluorophenyl)-3-[3-(trifluoromethyl)­phenyl]­thiourea and 1-(3-bromophenyl)-3-[3-(trifluoromethyl)-phenyl]­thiourea compounds effectively inhibited biofilm formation of methicillin-resistant and standard *S. epidermidis* strains.^42^ A study using 1-(2,5-Dichlorophenyl)-2-thiourea (DCPT) investigated the effects of different DCPT concentrations on biofilm formation by *S. aureus* and *E. coli*. They stated that as incubation time increased, the amount of biofilm formed by both *S. aureus* and *E. coli* increased, and a positive correlation was found between DCPT concentrations and the inhibition rate of biofilm formation.^43^ In a study in which a new series of thiourea derivatives bearing uracil ring were synthesized, it was reported that a weak effect on biofilm production was clearly seen in all compounds and in general, the activity against biofilm production of all potent compounds were weak, not exceeding 35% inhibition.^44^ Our study demonstrates that benzoylthiourea derivatives have significant potential in fighting infections caused by microorganisms like *S. aureus* and *P. aeruginosa*, which contribute to biofilm formation.

## 4 Conclusion

In this study, new perfluorinated benzoylthioureas (compounds 1 and 2) and their non-fluorinated analogue (3) were synthesized via the reaction of benzoyl isothiocyanate with the corresponding primary amines. The structures of the synthesized compounds were confirmed by FTIR and NMR spectroscopy. This study also focused on evaluating the biological activities of these compounds, including their antioxidant, antidiabetic, and DNA cleavage properties. The biological activities of benzoylthiourea derivatives, such as antimicrobial and antibiofilm properties, were also investigated in detail. *In vitro* assessments revealed that compounds 1 and 3 demonstrated notable DPPH scavenging activities. In addition, all compounds exhibited significant inhibitory potential against α-amylase. Furthermore, they exhibited a strong DNA cleavage effect that broke the DNA strand into small oligonucleotide fragments. The study’s findings revealed that the most resistant microorganisms to these compounds were *E. coli* as MIC values of 256 and 512 mg/L for 1 and 3, respectively and *P. aeruginosa* as MICs of 128 mg/L for 2, while the most sensitive ones were *Legionella pneumophila* subsp. *pneumophila* as MIC of 16, 16, and 32 mg/L for 1, 2, and 3, respectively and *E. faecalis* as MICs of 16, 8, and 32 mg/L for 1, 2, and 3, respectively. Moreover, it was determined that compounds containing fluorinated groups exhibited superior antimicrobial effects. As the concentration increased, the rate of inhibiting biofilm formation of *S. aureus* and *P. aeruginosa* increased significantly. At their highest tested concentrations, the benzoylthiourea derivatives (1, 2, and 3) demonstrated remarkable inhibitory effects on biofilm formation, achieving 71.79, 69.80, and 63.53% against *S. aureus*, respectively while against *P. aeruginosa*, they demonstrated significant inhibition of biofilm formation 53.52, 63.33, and 70%, respectively. The pharmacological activity of our thiourea compounds results from specific interactions between enzymes, proteins, and receptor targets. The protons on the two nitrogens act as hydrogen bond donors, while the C=S fragment of thiourea acts as a hydrogen bond acceptor. Also, the fluorinated group confers enhanced molecular stability and interactions. Therefore, the development of perfluorinated benzoylthiourea derivatives holds great potential for advanced research, offering valuable contributions to the literature on the design of innovative drugs and biological activities.

## Supplementary Material



## Notes

NMR and FTIR spectra for all compounds (PDF)
