# Supplementary material for: Design, Synthesis and Multitarget Biological Evaluation of Perfluoroalkylated Benzoylthiourea Compounds: From Biofilm Disruption to DNA Cleavage
Source: ACS Omega. 2026 Feb 9;11(7):11911–9. doi: 10.1021/acsomega.5c10893 (PMC12947037; doi:10.1021/acsomega.5c10893)
Supplement: Supplementary file 1 [file ao5c10893_si_001.pdf]

Design, Synthesis and Multitarget Biological Evaluation of Perfluoroalkylated Benzoylthiourea Compounds: From Biofilm Disruption to DNA Cleavage

Mustafa Kemal Yılmaz[a,b,*], Mustafa Kadir Esen[c], M. Serkan Yalçın[b,d], Simay İnce[b], Sadin Özdemir[b,c]

[a]*Department of Chemistry, Science Faculty, Mersin University, 33343, Mersin, TÜRKİYE*

[b]*Department of Nanotechnology and Advanced Materials, Institute of Science, Mersin University, 33343, Mersin, TÜRKİYE*

[c]*Food Processing Program, Technical Science Vocational School, Mersin University, 33343, Mersin, TÜRKİYE*

[d]*Department of Chemistry and Chemical Processing Technologies, Technical Science Vocational School, Mersin University, 33343, Mersin, TÜRKİYE*

*Email: mkyilmaz@mersin.edu.tr

Contents **Page**

Contents	Page
Figure S1: ^{1}H NMR spectrum of *N*-((4-(heptadecafluorooctyl)phenyl)carbamothioyl) benzamide (1)	S3
Figure S2: ^{13}C NMR spectra of *N*-((4-(heptadecafluorooctyl)phenyl)carbamothioyl) benzamide (1)	S3
Figure S3: ^{19}F NMR spectra of *N*-((4-(heptadecafluorooctyl)phenyl)carbamothioyl) benzamide (1)	S4
Figure S4: ^{1}H NMR spectrum of *N*-((3-(heptadecafluorooctyl)phenyl)carbamothioyl) benzamide (2)	S4
Figure S5: ^{13}C NMR spectra of *N*-((3-(heptadecafluorooctyl)phenyl)carbamothioyl) benzamide (2)	S5
Figure S6: ^{19}F NMR spectra of *N*-((3-(heptadecafluorooctyl)phenyl)carbamothioyl) benzamide (2)	S5
Figure S7: ^{1}H NMR spectrum *N*-(phenylcarbamothioyl)benzamide (3)	S6
Figure S8: ^{13}C NMR spectra of *N*-(phenylcarbamothioyl)benzamide (3)	S6
Figure S9: FTIR spectrum of *N*-((4-(heptadecafluorooctyl)phenyl)carbamothioyl) benzamide (1)	S7
Figure S10: FTIR spectrum of *N*-((3-(heptadecafluorooctyl)phenyl)carbamothioyl) benzamide (2)	S7
Figure S11: FTIR spectrum of *N*-(phenylcarbamothioyl)benzamide (3)	S8

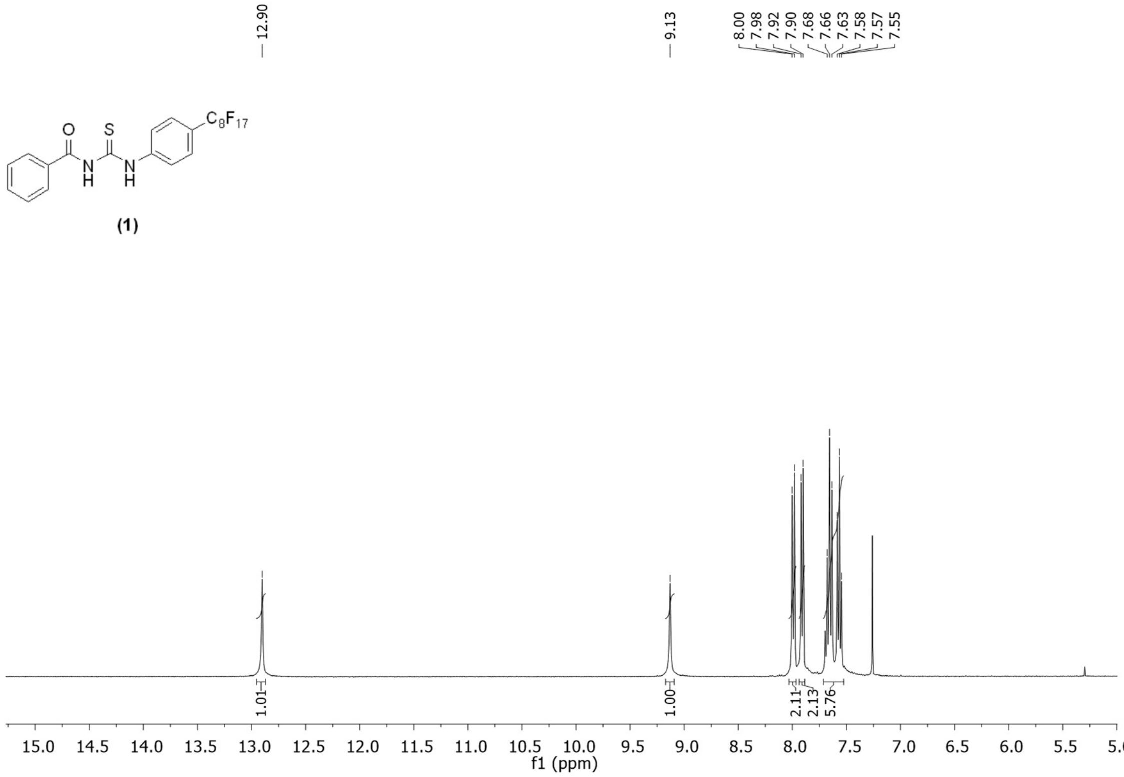

Figure S1: ^{1}H NMR spectrum of *N*-((4-(heptadecafluorooctyl)phenyl)carbamothioyl) benzamide (1)

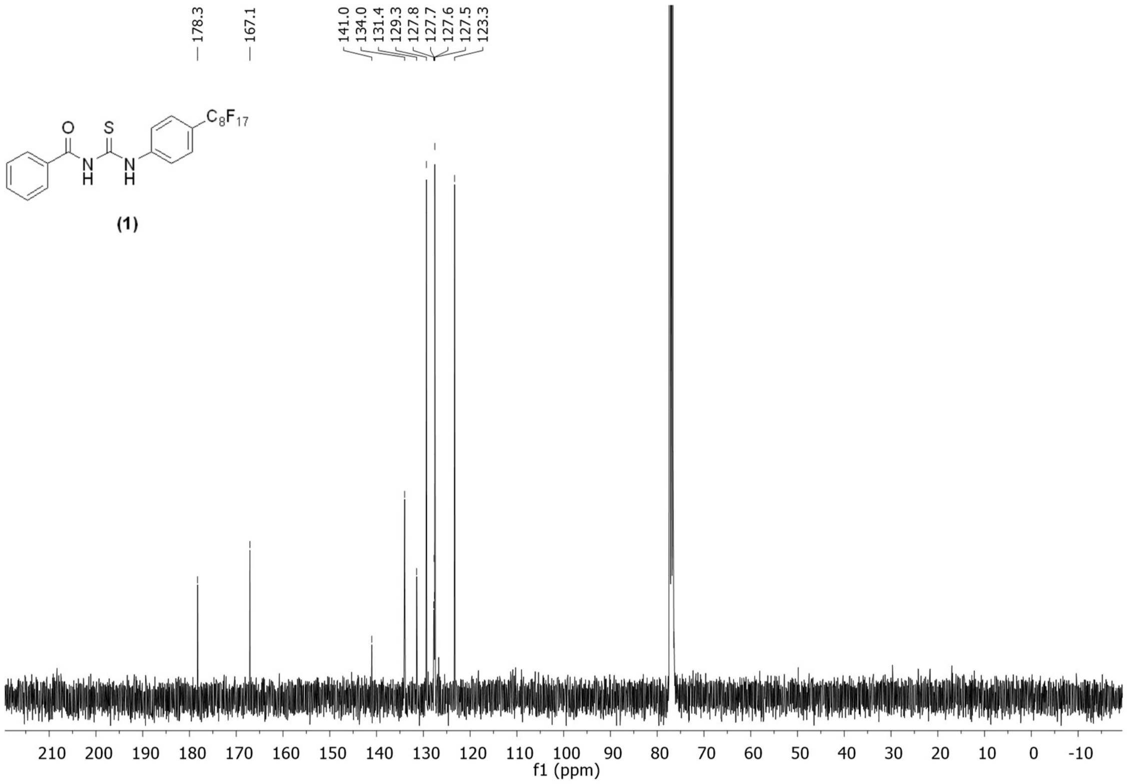

Figure S2: ^{13}C NMR spectra of *N*-((4-(heptadecafluorooctyl)phenyl)carbamothioyl) benzamide (1)

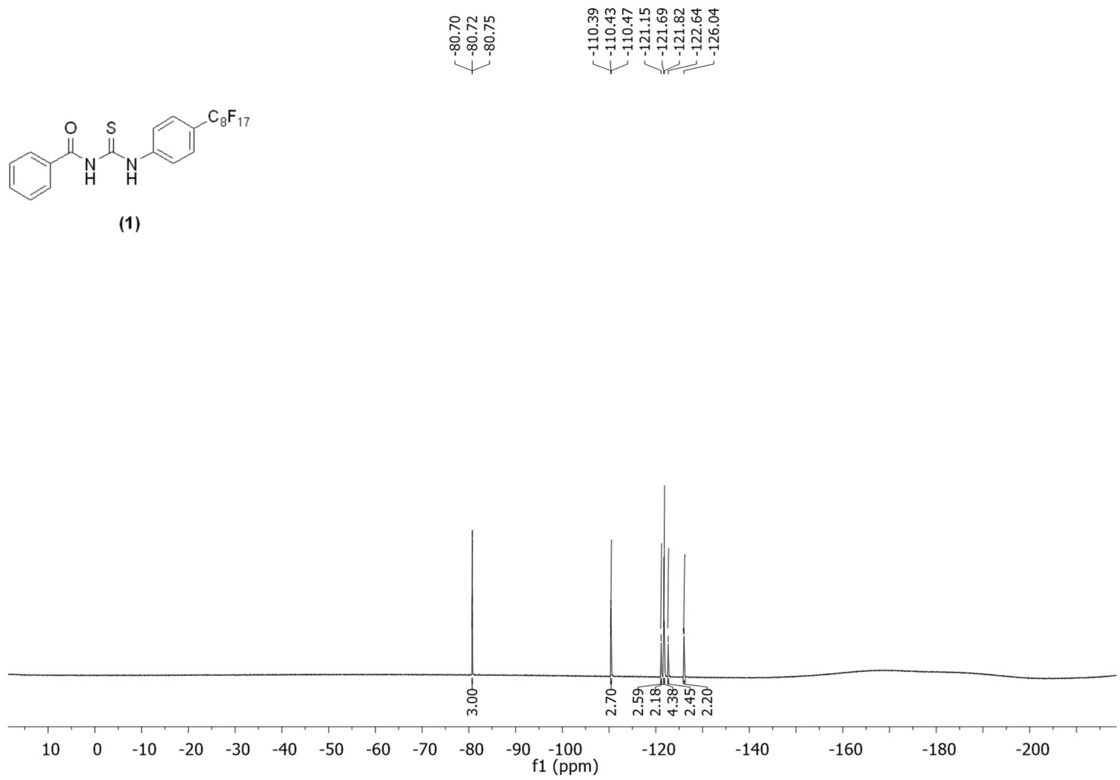

Figure S3: ^{19}F NMR spectra of *N*-((4-(heptadecafluorooctyl)phenyl)carbamothioyl) benzamide (1)

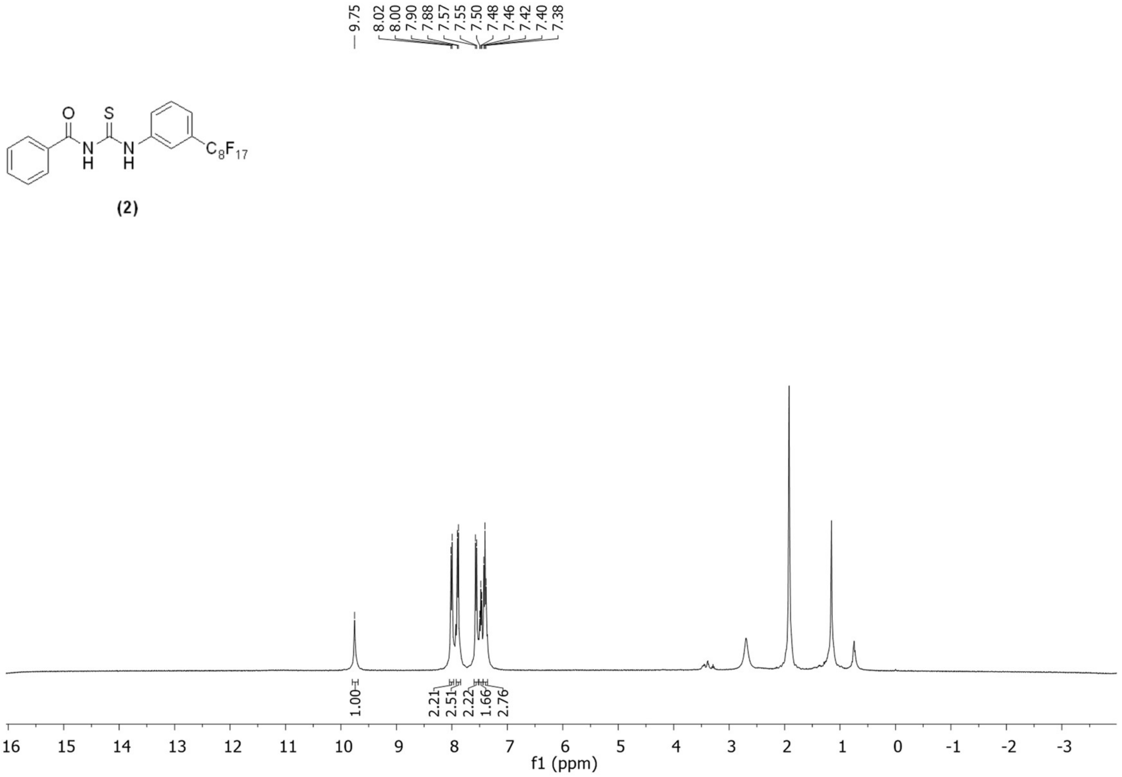

Figure S4: ^{1}H NMR spectrum of *N*-((3-(heptadecafluorooctyl)phenyl)carbamothioyl) benzamide (2)

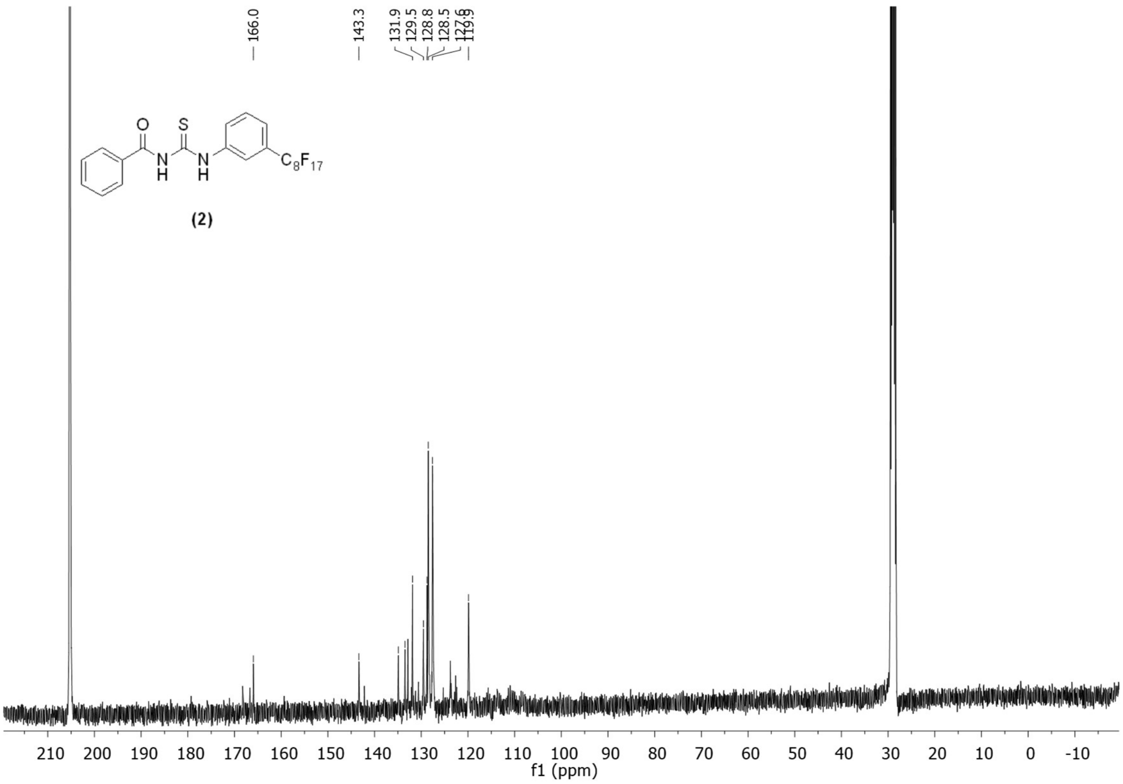

Figure S5: ^{13}C NMR spectrum of *N*-((3-(heptadecafluorooctyl)phenyl)carbamothioyl) benzamide (2)

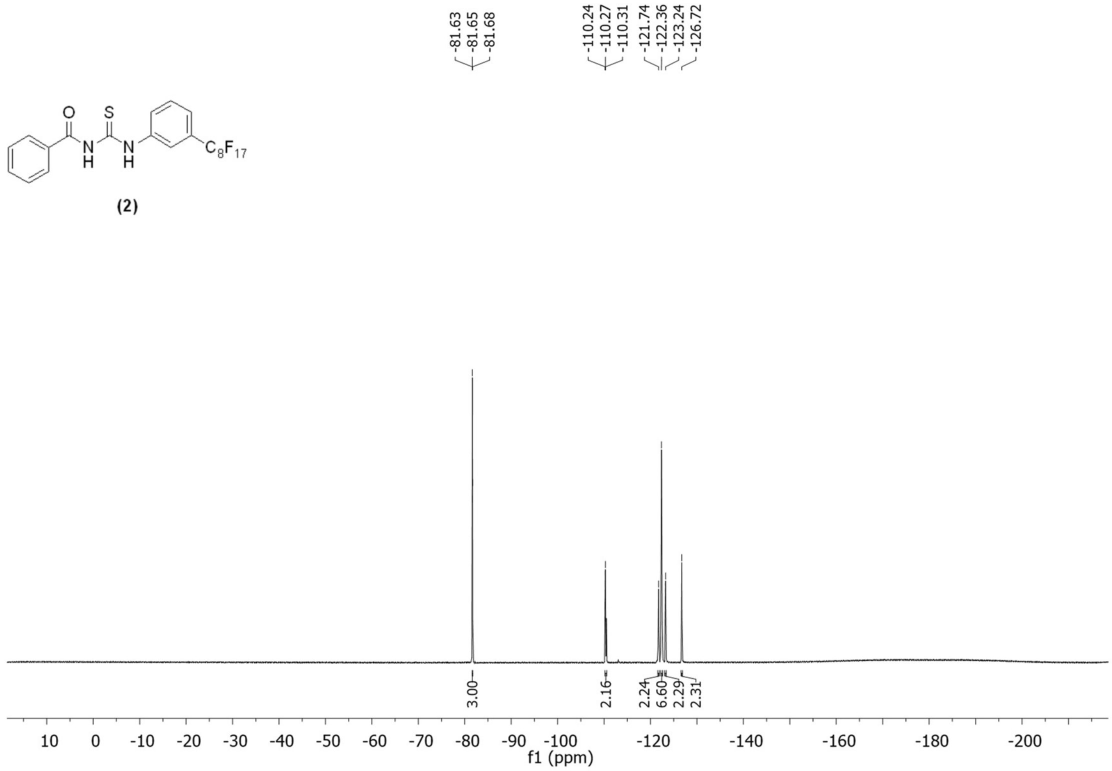

Figure S6: ^{19}F NMR spectrum of *N*-((3-(heptadecafluorooctyl)phenyl)carbamothioyl) benzamide (2)

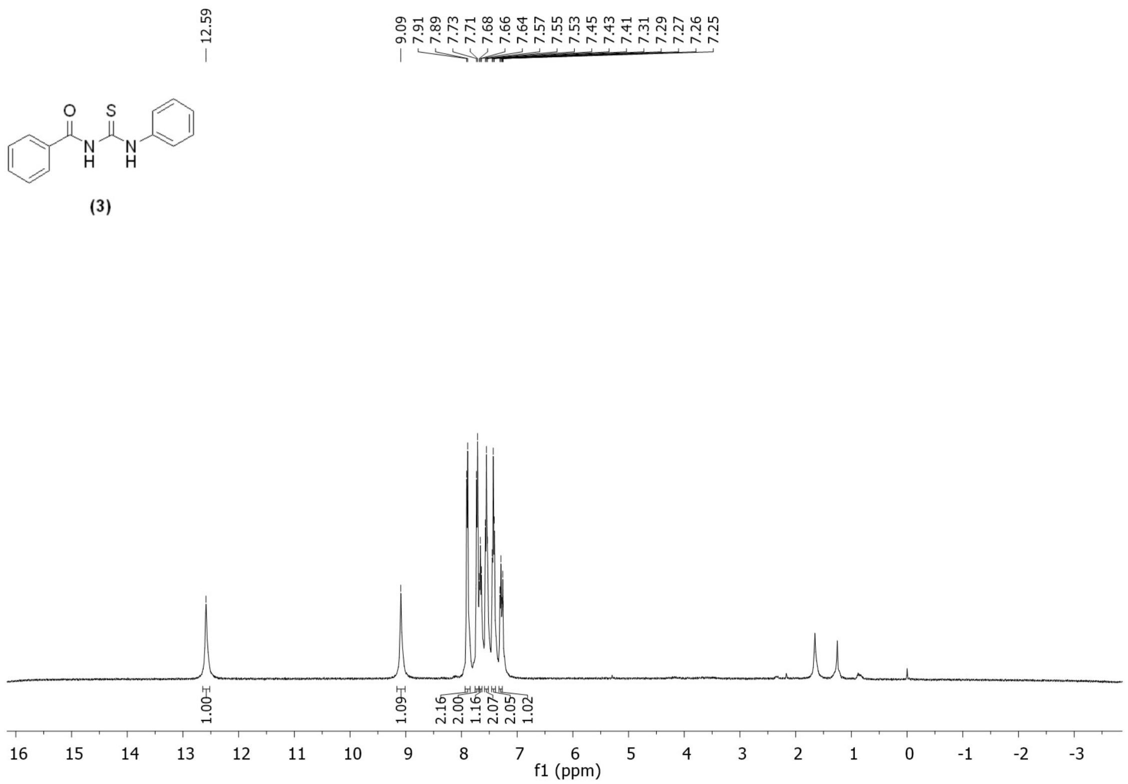

Figure S7: ^{1}H NMR spectrum *N*-(phenylcarbamothioyl)benzamide (3)

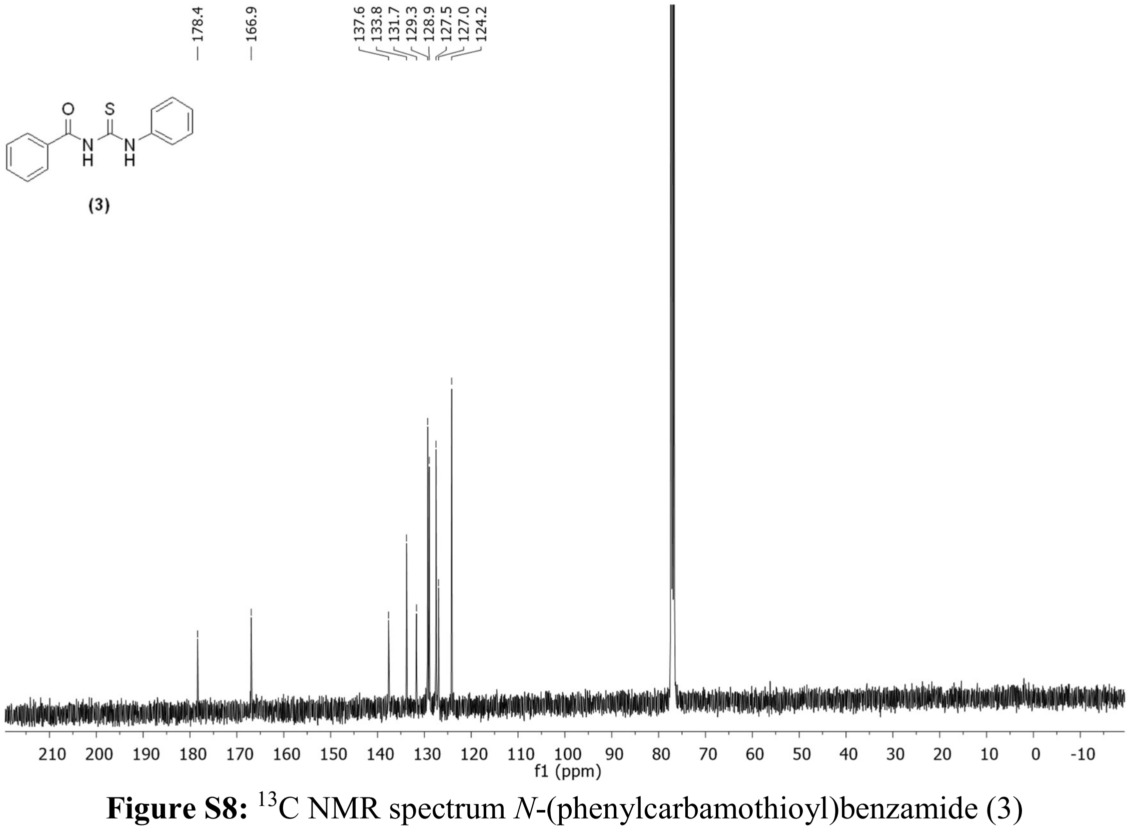

Figure S8: ^{13}C NMR spectrum *N*-(phenylcarbamothioyl)benzamide (3)

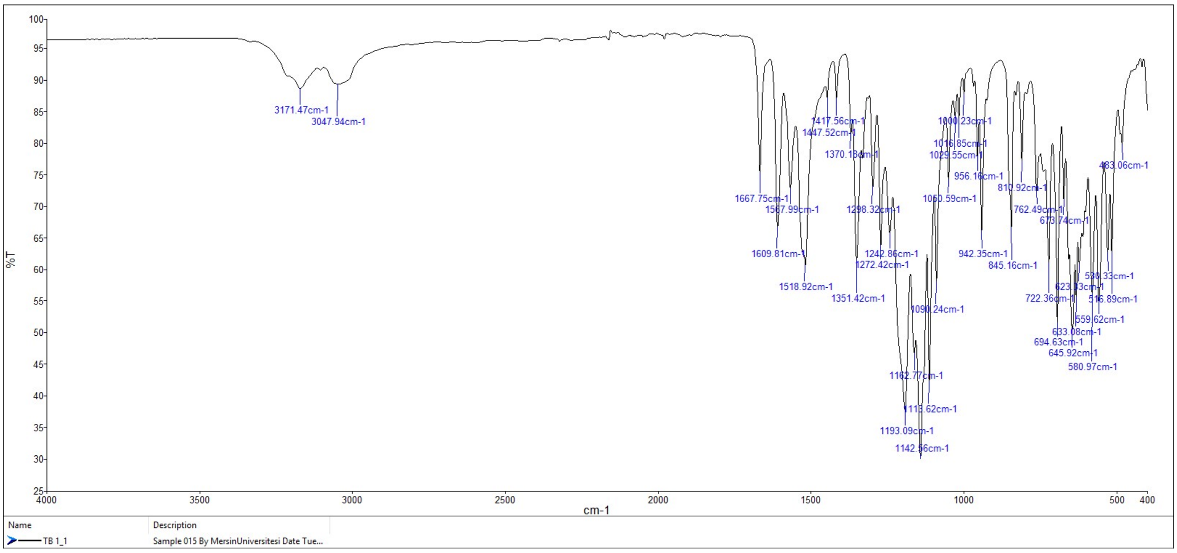

Figure S9: FTIR spectrum of *N*-((4-(heptadecafluorooctyl)phenyl)carbamothioyl) benzamide (1)

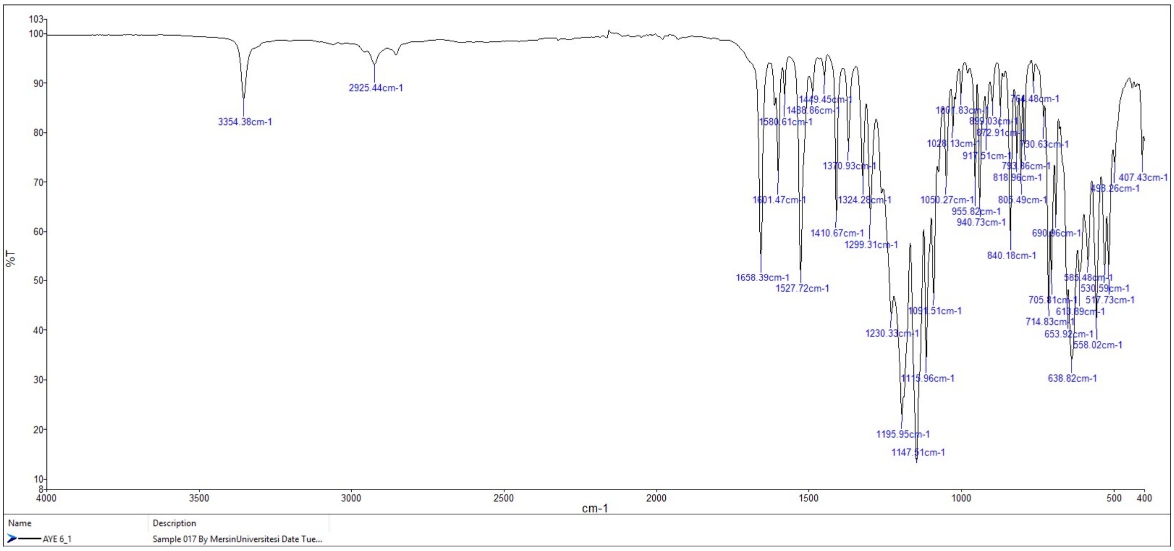

Figure S10: FTIR spectrum of *N*-((3-(heptadecafluorooctyl)phenyl)carbamothioyl) benzamide (2)

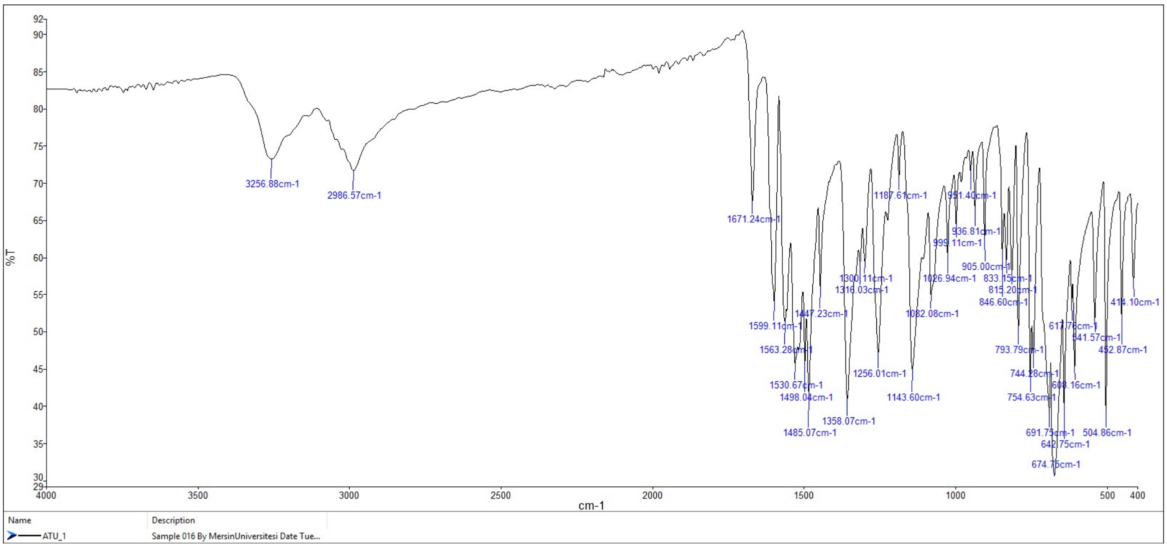

Figure S11: FTIR spectrum of *N*-(phenylcarbamothioyl)benzamide (3)
